# Haploidentical HSCT vs immunosuppressive therapy for severe aplastic anemia in patients aged 40-65 years: a retrospective comparative study

**DOI:** 10.1093/stcltm/szag076

**Published:** 2026-10-01

**Authors:** Sheng-nan Gao, Jin-song Jia, Xiao-jun Huang, Yuan-yuan Zhang, Ting-ting Han, Hai-xia Fu, Yun He, Yao Chen, Jing-zhi Wang, Xiao-dong Mo, Yu-qian Sun, Wei Han, Yu Wang, Xiao-hui Zhang, Lan-ping Xu, Zheng-li Xu

**Affiliations:** Peking University People’s Hospital, Peking University Institute of Hematology, National Clinical Research Center for Hematologic Disease, Beijing Key Laboratory of Cell and Gene Therapy for Hematologic Malignancies, Peking University, Beijing, China; Peking University People’s Hospital, Peking University Institute of Hematology, National Clinical Research Center for Hematologic Disease, Beijing Key Laboratory of Cell and Gene Therapy for Hematologic Malignancies, Peking University, Beijing, China; Peking University People’s Hospital, Peking University Institute of Hematology, National Clinical Research Center for Hematologic Disease, Beijing Key Laboratory of Cell and Gene Therapy for Hematologic Malignancies, Peking University, Beijing, China; Peking University People’s Hospital, Peking University Institute of Hematology, National Clinical Research Center for Hematologic Disease, Beijing Key Laboratory of Cell and Gene Therapy for Hematologic Malignancies, Peking University, Beijing, China; Peking University People’s Hospital, Peking University Institute of Hematology, National Clinical Research Center for Hematologic Disease, Beijing Key Laboratory of Cell and Gene Therapy for Hematologic Malignancies, Peking University, Beijing, China; Peking University People’s Hospital, Peking University Institute of Hematology, National Clinical Research Center for Hematologic Disease, Beijing Key Laboratory of Cell and Gene Therapy for Hematologic Malignancies, Peking University, Beijing, China; Peking University People’s Hospital, Peking University Institute of Hematology, National Clinical Research Center for Hematologic Disease, Beijing Key Laboratory of Cell and Gene Therapy for Hematologic Malignancies, Peking University, Beijing, China; Peking University People’s Hospital, Peking University Institute of Hematology, National Clinical Research Center for Hematologic Disease, Beijing Key Laboratory of Cell and Gene Therapy for Hematologic Malignancies, Peking University, Beijing, China; Peking University People’s Hospital, Peking University Institute of Hematology, National Clinical Research Center for Hematologic Disease, Beijing Key Laboratory of Cell and Gene Therapy for Hematologic Malignancies, Peking University, Beijing, China; Peking University People’s Hospital, Peking University Institute of Hematology, National Clinical Research Center for Hematologic Disease, Beijing Key Laboratory of Cell and Gene Therapy for Hematologic Malignancies, Peking University, Beijing, China; Peking University People’s Hospital, Peking University Institute of Hematology, National Clinical Research Center for Hematologic Disease, Beijing Key Laboratory of Cell and Gene Therapy for Hematologic Malignancies, Peking University, Beijing, China; Peking University People’s Hospital, Peking University Institute of Hematology, National Clinical Research Center for Hematologic Disease, Beijing Key Laboratory of Cell and Gene Therapy for Hematologic Malignancies, Peking University, Beijing, China; Peking University People’s Hospital, Peking University Institute of Hematology, National Clinical Research Center for Hematologic Disease, Beijing Key Laboratory of Cell and Gene Therapy for Hematologic Malignancies, Peking University, Beijing, China; Peking University People’s Hospital, Peking University Institute of Hematology, National Clinical Research Center for Hematologic Disease, Beijing Key Laboratory of Cell and Gene Therapy for Hematologic Malignancies, Peking University, Beijing, China; Peking University People’s Hospital, Peking University Institute of Hematology, National Clinical Research Center for Hematologic Disease, Beijing Key Laboratory of Cell and Gene Therapy for Hematologic Malignancies, Peking University, Beijing, China; Peking University People’s Hospital, Peking University Institute of Hematology, National Clinical Research Center for Hematologic Disease, Beijing Key Laboratory of Cell and Gene Therapy for Hematologic Malignancies, Peking University, Beijing, China

**Keywords:** severe aplastic anemia, haploidentical hematopoietic stem cell transplantation, immunosuppressive therapy, propensity score matching, elderly patients

## Abstract

Severe aplastic anemia (SAA) is a life-threatening hematological disorder, and the optimal treatment for patients aged ≥40 years remains controversial. This retrospective study aims to conduct a comparative analysis of the efficacy of immunosuppressive therapy (IST) versus haploidentical hematopoietic stem cell transplantation (haplo-HSCT) specifically in elderly patients. To minimize selection bias, propensity score matching (PSM) was performed to pair samples based on age and Eastern Cooperative Oncology Group score, thereby addressing treatment responses and survival outcomes while ensuring balanced baseline characteristics between the 2 groups. The study reveals that while overall survival (OS) rates were similar (69.6% ± 13.3% vs. 68.6% ± 12.1%, *P* = 0.80), haplo-HSCT significantly outperformed IST in terms of failure-free survival (FFS) rates (68.7% ± 12.1% vs. 46.5% ± 20.2%, *P* = 0.025). Multivariate Cox regression analysis confirmed that treatment modality and disease severity were independent predictors of FFS. These findings support haplo-HSCT as a viable first-line treatment option for appropriately selected elderly SAA patients, providing evidence for treatment options in patients with SAA aged 40 and above.

## Graphical Abstract

**Graphical Abstract:**
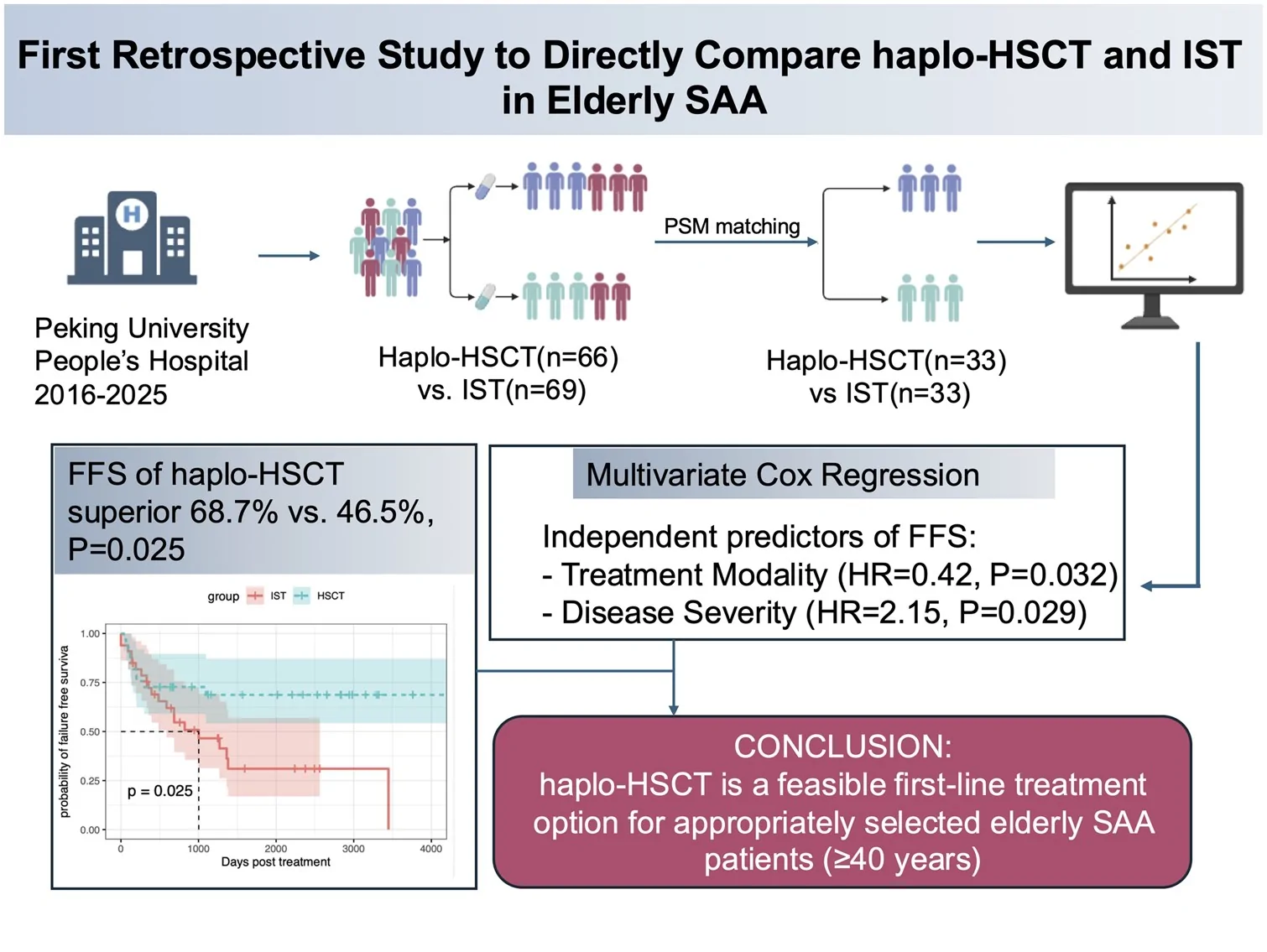

Significance statement This retrospective study addresses a critical gap in severe aplastic anemia (SAA) care for patients aged ≥40, where immunosuppressive therapy (IST) remains standard despite limitations. Using propensity score matching, it demonstrates that haploidentical hematopoietic stem cell transplantation (haplo-HSCT) yields superior failure-free survival versus IST, with comparable overall survival. These findings challenge age-based treatment dogma, validate haplo-HSCT as a viable first-line option for appropriately selected older SAA patients, and inform updated clinical guidelines to improve long-term outcomes in this underserved population.

## Introduction

Severe aplastic anemia (SAA) is a critical hematological disorder. Hematopoietic stem cell transplantation (HSCT) and immunosuppressive therapy (IST) are 2 effective options, decided based on patient age and disease status. According to the most recent British Society for Hematology (BSH) guidelines, for patients under 40 years with a matched related donor (MRD), HSCT is the first-line choice due to better failure free survival.^1^^,^^2^ For those patients over 40 years, IST remains the recommended first-line treatment. This recommendation is largely based on historical data showing increased transplant-related mortality (TRM) in older patients undergoing conventional myeloablative conditioning. Despite significant refinements in IST regimens over the past 2 decades, including the addition of eltrombopag and improved supportive care,^3^^,^^4^ substantial limitations persist. Long-term clonal evolution to myelodysplastic syndrome (MDS) or acute myeloid leukemia (AML), occurring in 10%-20% of patients; and a relapse rate of approximately 30%-40%. Consequently, long-term failure-free survival (FFS) with IST remains suboptimal, particularly in older adults with comorbidities.^5^

In parallel, the therapeutic landscape of allo-HSCT for SAA has evolved dramatically.^6^ Recent international studies have further optimized and validated the application of haploidentical HSCT in SAA treatment.^7^ The multicenter prospective BMT CTN 1502 trial from North America confirmed that haploidentical bone marrow transplantation with post-transplant cyclophosphamide (PTCy)-based prophylaxis achieves favorable 1-year overall survival (OS) of 81% and minimal graft-versus-host disease (GVHD) for patients with refractory or relapsed SAA.^7^ Consistently, the large-scale European Society for Blood and Marrow Transplantation Severe Aplastic Anemia Working Party (EBMT SAAWP) registry data demonstrated the safety and feasibility of PTCy prophylaxis in SAA patients receiving haploidentical, matched sibling, and unrelated donor transplantation, while also revealing relatively higher non-relapse mortality and inferior survival outcomes in haploidentical cohorts compared with other donor types.^8^ Beyond these advances in transplant modality optimization, emerging evidence has consistently challenged the conventional age barrier for transplantation. A landmark prospective study demonstrated that first-line matched sibling donor (MSD) allo-HSCT in SAA patients over 40 years yielded superior FFS compared to IST, establishing proof-of-concept that carefully selected older patients can benefit from upfront transplantation.^9^^,^^10^ Another registry data from the Chinese Blood and Marrow Transplant Registry Group (CBMTRG) have shown that in patients aged 40-50 years with SAA, outcomes following haploidentical HSCT were comparable to those after MSD transplantation, with OS rates exceeding 80%.^9^ In terms of patients age, retrospective data analysis of the CBMTRG cohort indicates good long-term results of HSCT in elderly SAA adults regardless of donor type.^6^^,^^11^^,^^12^ These collective findings strongly suggest that donor source may not be the primary determinant of survival in carefully selected older patients.

Despite these emerging data, a critical knowledge gap remains regarding the optimal approach in the era of modern haploidentical HSCT. Therefore, we conducted this retrospective study focusing on SAA patients older than 40 years old. To balance the bias introduced by baseline differences, particularly age as the key variable of interest in our study, we performed propensity score matching (PSM) based on age and ECOG score, directly comparing the outcomes of IST versus haploidentical HSCT in patients with SAA aged 40 years and older. By elucidating the differences in outcomes between these 2 groups, the study seeks to provide contemporary, real-world evidence to help redefine the age threshold and selection criteria for upfront transplantation, ultimately informing treatment algorithms for this underserved patient population.

## Methods

This retrospective study was approved by the Ethics Committee of Peking University People’s Hospital. The requirement for written informed consent was waived by the institutional review board due to its retrospective design, and all clinical procedures were performed in accordance with the Declaration of Helsinki. After being counseled on the advantages and disadvantages of both treatment options, patient guardians selected either IST or haploidentical transplantation as first-line therapy.

### IST and evaluation

Sixty-six patients with SAA aged over 40 years (range, 40-78 years) receiving IST at Peking University People’s Hospital between August 2016 and April 2025 were initially enrolled (Table 1). After PSM with the haploidentical HSCT group, 33 patients aged 40-65 years remained for final analysis.

**Table 1.** Patient characteristics.

		All patients			Patients matched by propensity score		
		IST (*n* = 66)	HSCT (*n* = 69)	*P*	IST (*n* = 33)	HSCT (*n* = 33)	*P*
**Age, median (range)**		56 (40, 78)	44 (40, 64)	<.001	49 (40, 64)	47 (40, 64)	.396
**Gender (%)**	Male	26 (39.4)	41 (59.4)	.025	13 (39.4)	20 (60.6)	.139
	Female	40 (60.6)	28 (40.6)		20 (60.6)	13 (39.4)	
**Severity (%)**	SAA	53 (80.3)	52 (75.4)	.539	27 (81.8)	28 (84.8)	1.00
	vSAA	13 (19.7)	17 (24.6)		6 (18.2)	5 (15.2)	
**Disease course, months, (median [IQR])**		2 [1, 2]	24 [8, 168]	<.001	2 [1, 2]	24 [11, 120]	<.001
**ECOG (%)**	0	18 (27.3)	19 (27.5)	<.001	14 (42.4)	8 (24.2)	.17
	1	22 (33.3)	49 (71.0)		16 (48.5)	24 (72.7)	
	2	26 (39.4)	1 (1.4)		3 (9.1)	1 (3.0)	
**Treatment year (%)**	2021-2025	43 (65.2)	42 (60.9)	.722	19 (57.6)	25 (75.8)	.191
	2016-2020	23 (34.8)	27 (39.1)		14 (42.4)	8 (24.2)	
**Alive (%)**		43 (65.2)	51 (73.9)	.349	22 (66.7)	23 (69.7)	1.00

Abbreviations: ECOG, Eastern Cooperative Oncology Group Score; HSCT, hematopoietic stem cell transplantation; IQR, interquartile range; IST, immunosuppressive therapy; SSA, severe aplastic anemia; vSAA, very SAA.

The IST protocol comprised: rabbit ATG (Sangstat, Lyon, France) 3-4 mg/kg/day intravenously for 5 consecutive days; prednisone 1 mg/kg/day for 10 days (initiated before first ATG dose) with rapid taper over 1-2 weeks; cyclosporine (CsA) 3-5 mg/kg/day orally twice daily for 6 months or until plateau response, then continued for 12-24 months before discontinuation; dose-adjusted to maintain 150-250 μg/L levels.

Hematologic response was assessed at 3 and 6 months post-ATG + CsA. A complete response (CR) was defined as meeting all 3 peripheral blood count criteria: (1) neutrophil count >1.5 × 10⁹/L, (2) hemoglobin level >110 g/L, and (3) platelet count >100 × 10⁹/L. A partial response (PR) was defined as neutrophil count >0.5 × 10⁹/L, hemoglobin level >80 g/L, platelet count >20 × 10⁹/L, and transfusion independence.^13^^,^^14^

### Haploidentical transplantation

Sixty-nine SAA/very SAA (vSAA) patients aged over 40 years (range, 40-65 years) receiving haploidentical transplantation at Peking University People’s Hospital between August 2016 and April 2025 were initially enrolled; all met the diagnostic criteria for severe or very SAA. After PSM with the IST cohort, 33 patients aged 40-65 years were retained for final analysis.

The haploidentical SCT conditioning regimen consisted of intravenous busulfan (0.8 mg/kg 4 times daily, days −8 to −7), cyclophosphamide (25 mg/kg daily, days −5 to −2), fludarabine (Flu) 30 mg/m^2^ days −6 to −2, and rabbit-ATG (2.5 mg/kg daily, days −5 to −2). Stem cell source was G-CSF-primed bone marrow combined with peripheral blood. GVHD prophylaxis included CsA, mycophenolate mofetil, and short-course methotrexate. Additional procedural details have been previously reported.^15–18^

### Definitions

vSAA or SAA was diagnosed according to guidelines.^19^ OS was defined as the interval from treatment initiation to death. Performance status prior to transplantation and at the last follow-up was assessed using the Eastern Cooperative Oncology Group (ECOG) scale.

In the HSCT group, neutrophil and platelet engraftment was defined as previous report.^15^ Failures after HSCT was defined as death, primary and secondary graft failure and relapse, whichever occurred first. Primary graft failure (GF) was defined as failure of myeloid engraftment until 28 days after transplantation. Secondary GF was defined as absence of graft function after full engraftment. Acute and chronic GVHD (aGVHD and cGvHD) were graded using standard criteria.^19–21^ Hematopoietic Cell Transplantation Comorbidity Index (HCT-CI) used to assess transplant risk.^22^^,^^23^ Epstein-Barr virus (EBV) DNAemia > 400 copies/mL, cytomegalovirus (CMV) > 1000 copies/mL were the thresholds for positive results.

In the IST group, IST treatment failure encompassed death, absence of response at or beyond 6 months, disease progression necessitating intervention, relapse, and clonal evolution.

### Statistical analysis

Patient characteristics were compared using chi-square or Fisher’s exact tests for categorical variables, and the Mann–Whitney *U* test for continuous variables. OS and FFS were measured using the Kaplan–Meier estimator and compared using the log-rank test. The Univariate and multivariate analyses were performed using a Cox proportional hazard regression model. Univariate and multivariate analysis were performed using a Cox proportional hazard regression model. The statistical analyses were performed under R version 4.4.1. *P* < .05 was considered statistically significant; *P < .05, **P < .01, ***P < .001.

## Results

### Patients characteristics

The demographic characteristics of the IST and HSCT groups are presented in Table 1. The 2 groups were comparable in age, gender, disease severity, year of treatment, ECOG score and survival rate distribution. However, significant differences were observed in disease duration before treatment. The HSCT group had significantly longer disease duration than the IST group (a median 24 months vs. 2 months before treatment), 30 of 33 (90.9%) patients had disease duration longer than 4 months. Moreover, in the HSCT cohort, 6 of 33 (18.2%) patients had experienced IST failure (Table 2), and haplo-HSCT served as second-line therapy for these patients.

**Table 2.** Patients and transplant characteristics in the HSCT group.

Variable	Level	Statistic
**ATG pre-HSCT, *n* (%)**	No	27 (81.8)
	Yes	6 (18.2)
**HCT-CI, *n* (%)**	0	12 (36.4)
	1	12 (36.4)
	2	6 (18.2)
	3	2 (6.1)
	4	1 (3.0)
**MNCs, ×10^8^, median [IQR]**		9.36 [8.38, 12.50]
**CD34+, ×10^6^, median [IQR]**		3.38 [2.34, 4.77]
**Donor selection, *n* (%)**	Parents	1 (3.0)
	Sibling	10 (30.3)
	Child	22 (66.7)
**Donor-recipient gender, *n* (%)**	Male–male	18 (54.5)
	Male–female	6 (18.2)
	Female–Female	7 (21.2)
	Female–male	2 (6.1)
**ABO match, (%)**	Match	15 (45.5)
	Minor mismatch	10 (30.3)
	Major mismatch	4 (12.1)
	Bidirectional mismatch	4 (12.1)
**Neutrophil engraftment, days, median [IQR]**		12 [11, 16]
**Platelet engraftment, days, median [IQR]**		16 [11, 23]
**aGVHD within 100 days, *n* (%)**	No	25 (75.8)
	Yes	7 (21.2)
**aGVHD grade (%)**	0	26 (78.8)
	I	3 (9.1)
	II	3 (9.1)
	III-IV	1 (3.0)
**cGVHD, *n* (%)**	No	21 (63.6)
	Yes	2 (6.1)
**cGVHD grade (%)**	0	31 (93.9)
	I	2 (6.1)
**Graft failure (%)**		0
**CMV (%)**	No	15 (45.5)
	Yes	16 (48.5)
**CMV positive time point, days, median [IQR]**		37 [26, 40]
**EBV (%)**	No	23 (69.7)
	Yes	8 (24.2)
**EBV positive time point, days, median [IQR]**		41 [37, 49]

Abbreviations: aGvHD, acute graft-versus-host disease; cGvHD, chronic graft-versus-host disease; CMV, cytomegalovirus; EBV, Epstein-Barr Virus; HCT-CI, Hematopoietic Cell Transplantation Comorbidity Index; HSCT, hematopoietic stem cell transplantation; IQR, interquartile range.

### Outcomes of IST

Patient and treatment characteristics of the IST group are shown in Table 3. Among the 33 patients in the IST group, 100% patients underwent efficacy evaluation at 3 months post-treatment. Complete remission was achieved in 2 patients (6.1%), partial remission in 19 patients (57.6%), and no response in 12 patients (36.4%). At the 6-month time point, 1 patient (3%) had died within 6 months, 3 patients (9.1%) achieved complete remission, 21 patients (63.6%) achieved partial remission, 8 patients (24.2%) had no remission, and 11 patients (33.3%) relapsed after achieving remission with a median time to relapse of 2.25 years. A total of 3 patients in the IST group underwent HSCT after treatment failure.

**Table 3.** Patient and treatment characteristics of the IST group.

Variable	Level	Statistic
**3 months evaluation, *n* (%)**	CR	2 (6.1)
	PR	19 (57.6)
	NR	12 (36.4)
**6 months evaluation, *n* (%)**	CR	3 (9.1)
	PR	21 (63.6)
	NR	8 (24.2)
	Dead	1 (3.0)
**Adverse reactions, *n* (%)**	No	19 (57.6)
	ATG-related	2 (6.1)
	Hemorrhage-related	2 (6.1)
	Infection-related	10 (30.3)

Abbreviations: CR, complete response; PR, partial response; NR, non-response.

During the initial 6 months of treatment, the incidence of ATG-related adverse reactions was 6.1%, hemorrhage-related adverse reactions was 6.1%, and infection-related adverse reactions was 30.3%.

### Outcomes of HSCT

Descriptions of patients and transplant characteristics in the HSCT group are provided in Table 2. Among the 33 patients in the HSCT group, 1 patient died on day 4 post-infusion due to sudden cardiac death attributed to conditioning regimen-related cardiotoxicity, apart from this patient, all others achieved successful myeloid engraftment, with median neutrophil and platelet engraftment times of 12 and 16 days, respectively. Six patients (18.2%) had received prior IST before transplantation, and nine patients (27.3%) had pretransplant HCT-CI scores of 2 or higher.

aGVHD occurred in 7 of 33 patients, with 3 patients at grade I, 3 patients at grade II, and 1 patient at grade III-IV. As of the follow-up date, cGVHD developed in 2 surviving patients (6.1%), both of whom had limited involvement.

The 33 patients in the HSCT group achieved a 96.97% ± 3.03% cumulative engraftment rate. The cumulative incidence of CMV infection at day 100 was 48.48% ± 8.89%, with a median time to infection of 37 days post-transplantation. The cumulative incidence of EBV infection at day 100 was 24.24% ± 7.6%, with a median time to infection of 41 days post-transplantation. Among patients with CMV viremia, 2 progressed to clinical CMV disease. One patient developed CMV encephalitis and recovered completely after standardized antiviral therapy. The other patient presented with CMV pneumonia accompanied by mixed bacterial and fungal infections, and succumbed to infectious toxic shock. In terms of EBV viremia, a total of 3 patients developed PTLD, consisting of 2 cases of clinically suspected PTLD and 1 case of pathologically confirmed PTLD. Both patients with clinically suspected PTLD achieved clinical remission after targeted treatment; however, 1 patient died of severe gastrointestinal hemorrhage during follow-up. The patient with pathologically confirmed PTLD showed progressive disease despite intervention, and eventually died secondary to Gram-negative bacillary infectious toxic shock.

### Survival outcomes

Median follow-up was 850 days for the IST cohort and 1122 days for the haploidentical HSCT cohort. After PSM, the 3-year OS from treatment start was estimated at 69.6% ± 13.3% in the IST group versus 68.6% ± 12.1% in the HSCT group (Figure 1, *P* = .80). The corresponding 3-year FFS was 46.5% ± 20.2% for IST and 68.7% ± 12.1% for HSCT (Figure 2, *P* = .025). Prior to PSM, the 3-year OS was 73.6% ± 8.6% in the IST group and 73.3% ± 7.4% in the HSCT group (Figure S1, *P* = .28). The pre-matching 3-year FFS was 49.6% ± 13.6% for IST versus 73.3% ± 7.4% for HSCT (Figure S2, *P* < .0001).

**Figure 1.**
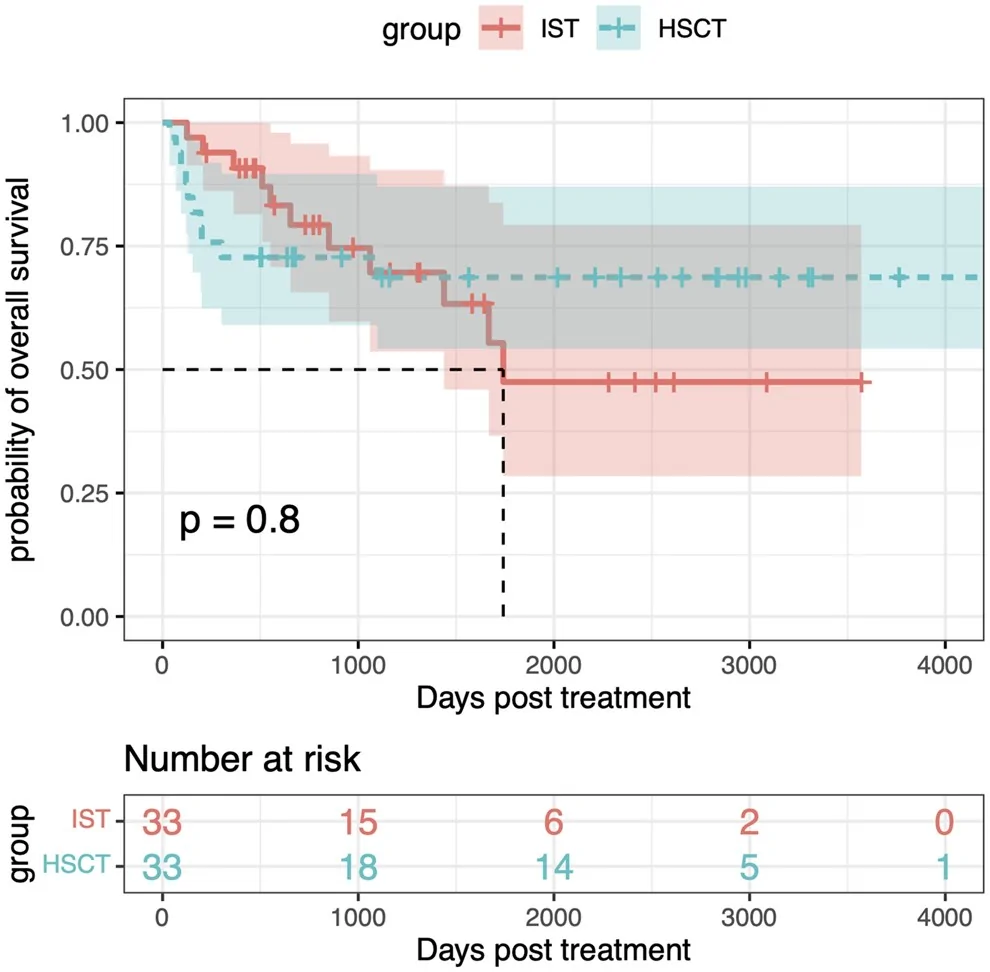
Overall survival of IST versus haplo-HSCT cohort after propensity score matching (*n* = 33 vs. 33, *P* = 0.8).

**Figure 2.**
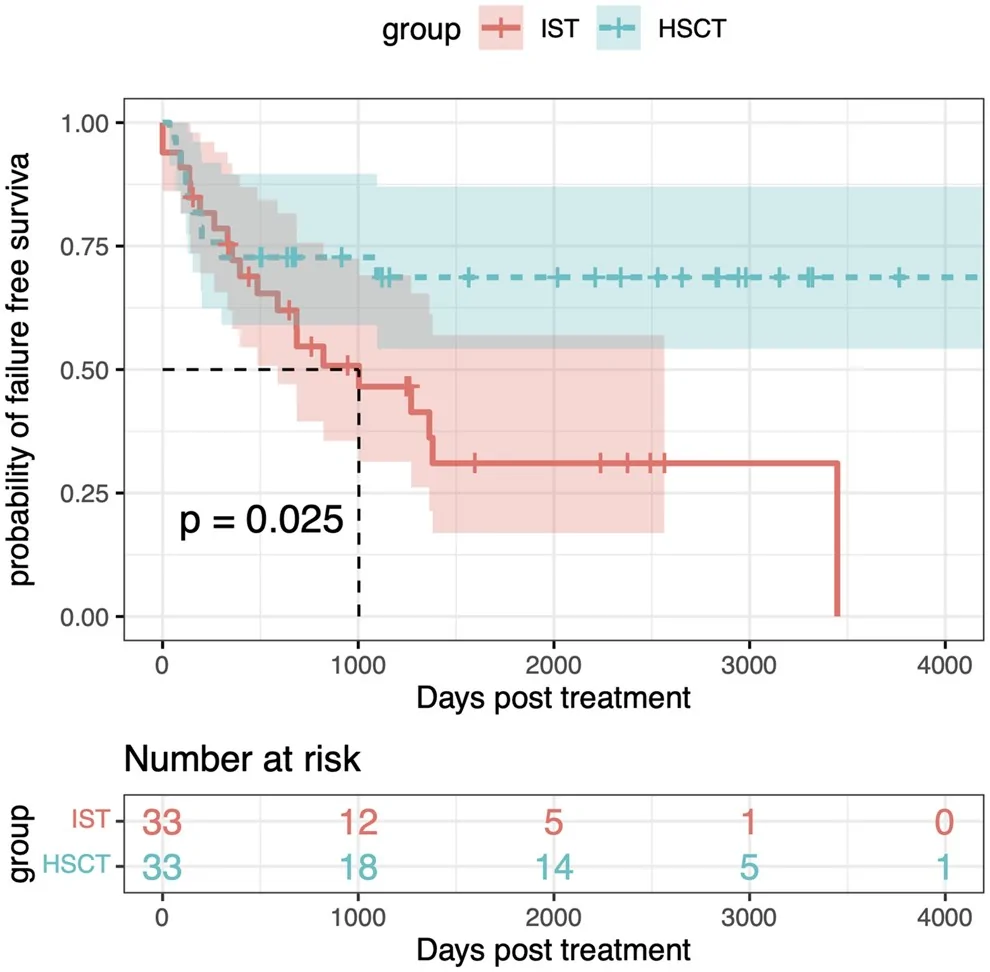
Failure free survival of IST versus haplo-HSCT cohort after propensity score matching (*n* = 33 vs. 33, *P* = 0.025). haplo-HSCT, haploidentical hematopoietic stem cell transplantation; IST, immunosuppressive therapy.

We subsequently performed univariate(Table 4) and multivariate Cox regression analyses (Table 5) for OS and FFS. The results demonstrated that in univariate analysis, variables significantly influencing OS included age (HR = 1.1, *P* = .029) and disease severity (HR = 4.1, *P* = .0017), while those significantly influencing FFS encompassed treatment modality (HR = .43, *P* = .029), age (HR = 1.1, *P* = .00051) and disease severity (HR = 4.1, *P* = .0017) (Table 4). To further adjust for the influence of confounding factors, multivariate analysis revealed that only disease severity (HR = 5.15, *P* = .001) remained significantly associated with OS, whereas both treatment modality and disease severity (HR = 5.39, *P* = .0003) were significantly associated with FFS. The hazard ratio for the effect of treatment modality on FFS was .39 (*P* = .03) (Table 5), indicating that HSCT was associated with significantly improved treatment outcomes.

**Table 4.** Factors associated with outcomes in univariate analysis.

Variable	Overall survival		Failure-free survival	
	HR (95% CI)	*P*	HR (95% CI)	*P*
**Group**	0.89 (0.38-2.1)	.8	0.43 (0.2-0.92)	**.029**
**Age**	1.1 (1-1.2)	**.029**	1.1 (1.1-1.2)	**.00051**
**Gender**	0.87 (0.37-2.1)	.75	0.93 (0.45-1.9)	.85
**Severity**	4.1 (1.7-10)	**.0017**	3 (1.3-6.9)	**.0076**
**Disease course**	1 (0.99 -1)	.4	0.99 (0.99-1)	.12
**ECOG**	1.3 (0.82-2)	.27	1.3 (0.64-2.5)	.51
**Treatment year**	1.6 (0.64-4.2)	.31	1.9 (0.83-4.2)	.13

Abbreviation: ECOG, Eastern Cooperative Oncology Group Score.Values in bold denote statistically significant results with *P*‑value < 0.05.

**Table 5.** Multivariate analysis of adverse factors associated with survival outcomes.

Variable	Overall survival		Failure-free survival	
	HR	*P*	HR	*P*
**Group**	1.20	.73	0.39	**.03**
**Age**	1.09	.12	1.08	.06
**Severity**	5.15	**.001**	5.39	**.0003**
**ECOG**	0.86	.73	1.29	.48
**Treatment year**	1.71	.36	2.75	.04

Abbreviation: ECOG, Eastern Cooperative Oncology Group Score.Values in bold denote statistically significant results with *P*‑value < 0.05.

## Discussion

The management of SAA in patients aged 40 years and older represents a considerable therapeutic challenge, characterized by a delicate balance between competing risks. On one hand, IST aims at modulating the aberrant immune response that suppresses hematopoiesis but carries a substantial risk of relapse and clonal evolution, threatening long-term survival. On the other hand, HSCT has the potential to restore normal hematopoiesis but is limited by TRM due to diminished organ function reserve and increased comorbidities in the elderly patients.^24^ These challenges underscore the critical need for comparative evaluations of treatment modalities tailored to elderly patients to optimize clinical decision-making and improve outcomes. To our knowledge, this is the first retrospective study to directly compare haploidentical HSCT with IST in these specific elderly patient population.

In the treatment of SAA, rapid hematopoietic reconstitution is an advantage of HSCT, which is conent with our study and previous reports.^9^^,^^25^^,^^26^ In our analysis, the complete remission rate at 3 months in the IST group was only 6.1%, and at 6 months it was merely 9.1%. In the haploidentical transplantation group, our transplantation cohort demonstrated robust outcomes, 96.97% ± 3.03% patients achieved successful engraftment, with acceptable and manageable rates of both acute and chronic GVHD and viral reactivation, the median times to neutrophil and platelet engraftment were 12 days and 16 days, respectively, with significant differences in FFS. Therefore, haploidentical HSCT is associated with significantly faster hematopoietic recovery and superior FFS, suggesting that age alone should no longer be the sole determinant in treatment selection.

Previous studies in younger SAA patients have demonstrated that HSCT is superior to IST in terms of long-term FFS.^2^^,^^25–27^ Due to recent advances and optimization of transplantation techniques, we have reached the same conclusion in patients over 40 years of age. In recent years, remarkable advances in conditioning regimen optimization and GVHD prophylaxis strategies have substantially expanded the therapeutic ceiling of allo-HSCT for aplastic anemia. Notably, haploidentical transplantation has progressed at an unprecedented pace. A landmark prospective cohort study provided the first definitive evidence that first-line MSD HSCT yielded superior long-term FFS compared to IST in older patients, challenging decades of established practice.^26^ Subsequently, a propensity score-matched analysis in Cancer Letters demonstrated equivalent outcomes between MSD and haploidentical HSCT in patients over 40, with survival rates exceeding 80% in both groups.^9^ Further supporting this paradigm shift, a registry-based study illustrated the feasibility and efficacy of transplantation in patients over 50 years using both haploidentical and matched donors.^10^ These cumulative findings have prompted Chinese consensus guidelines to recommend HSCT as a viable first-line option for patients up to 50 years of age. Building upon this evidence base, our single-center retrospective analysis provides timely, real-world comparative data to further refine treatment algorithms.

In our IST cohort, the 6-month overall response rate was 72%, which is slightly higher than the data from the French Severe Aplastic Anemia Reference Center registry, attributable to the recent addition of eltrombopag (EPAG) in IST. However, the relapse rate remained high, with a failure event rate of 66.7%, significantly higher than the 33.3% observed in the HSCT cohort. The predominant causes of mortality in this group were hemorrhagic complications and infections, directly attributable to the slow and often incomplete hematologic recovery inherent to IST. In our haploidentical transplantation cohort, over 90% of patients received CsA-based treatment before transplantation, resulting in longer disease duration. The total disease duration of included patients was significantly longer than that in the IST cohort (median disease duration 24 vs 2 months), and 18.2% of patients had experienced failed IST prior to transplantation, both of which are adverse prognostic factors for SAA treatment. Nevertheless, the outcomes remained favorable, these factors did not affect its advantage. Successful engraftment was achieved among all alive patients, with acceptable and manageable rates of both acute and chronic GVHD and viral reactivation.

From baseline characteristics, the HSCT group had longer disease duration, these mismatched baseline features may introduce result bias. However, we employed multivariate analysis to eliminate the impact of mismatched baseline characteristics. After matching for age and ECOG score as PSM pairing factors and incorporating variables with significant risk in univariate analysis, multivariate analysis revealed that treatment modality and disease severity remained key variables affecting FFS. The long-term FFS was remarkably promising, supporting the viability of this approach in appropriately selected patients.

We acknowledge several limitations in our study. Patients in the HSCT group had significantly longer disease duration than those in the IST group, and transplantation was not first-line therapy, suggesting worse organ reserve function in the HSCT group. Encouragingly, the impact of prolonged disease duration was offset by the treatment modality. Nevertheless, while the retrospective cohort design allows for the analysis of existing patient data from 1 single center, which may introduce bias in treatment allocation and patient outcomes. The relatively small sample size limits the generalizability of our findings to the broader elderly population suffering from SAA. Prospective, multicenter validation studies are needed to confirm these findings and establish clear selection criteria.

In summary, our study represents the first retrospective analysis comparing haploidentical HSCT with IST specifically in patients with SAA aged 40 years and older. We demonstrated that haploidentical HSCT was associated with faster hematopoietic reconstitution and significantly improved FFS. These results provide compelling clinical evidence to support the consideration of haploidentical transplantation as a preferred therapeutic option for elderly patients with SAA, potentially redefining the age-based treatment paradigm in the modern era of transplantation. Nevertheless, several inherent limitations of this retrospective work should be acknowledged when interpreting our findings. As a single-center non-randomized investigation, residual confounding effects may persist even after PSM. The notable disparity in disease duration between cohorts may introduce selection bias. Moreover, the moderate sample size of 33 patients per group limits statistical power for subgroup analyses and constrains the broader generalizability of our observations.

## Supplementary Material

szag076_Supplementary_Data
