# Supplementary material for: Haploidentical HSCT vs immunosuppressive therapy for severe aplastic anemia in patients aged 40-65 years: a retrospective comparative study
Source: Stem Cells Transl Med. 2026 Oct 1;15(10):szag076. doi: 10.1093/stcltm/szag076 (PMC13629439; doi:10.1093/stcltm/szag076)
Supplement: Supplementary file 1 [file szag076_supplementary_data.pdf]

Supplementary Information

Haploidentical HSCT vs Immunosuppressive Therapy for Severe Aplastic Anemia in Patients Aged 40-65 Years: A Retrospective Comparative Study ---- Haplo-HSCT vs IST for 40–65 Year SAA Patients

Authors: Sheng-nan Gao[1#], Jin-song Jia[1#], Xiao-jun Huang[1], Yuan-yuan Zhang[1], Ting-ting Han[1], Hai-xia Fu[1], Yun He[1], Yao Chen[1], Jing-zhi Wang[1], Xiao-dong Mo[1], Yu-qian Sun[1], Wei Han[1], Yu Wang[1], Xiao-hui Zhang[1], Lan-ping Xu [1*], Zheng-li Xu [1*]

[#] These authors contributed equally to this work as first authors.

* These authors contributed equally to this work as corresponding authors.

Author Affiliations

1 Peking University People's Hospital, Peking University Institute of Hematology, National Clinical Research Center for Hematologic Disease, Beijing Key Laboratory of Cell and Gene Therapy for Hematologic Malignancies, Peking University, Beijing, China

Corresponding author

Peking University People's Hospital, No. 11, Xizhimen South Street, Xicheng District, Beijing 100044, China.

Zheng-li Xu, Email: xuzhengli0202@163.com

Lan-ping Xu, Email: lpxu_0415@sina.com

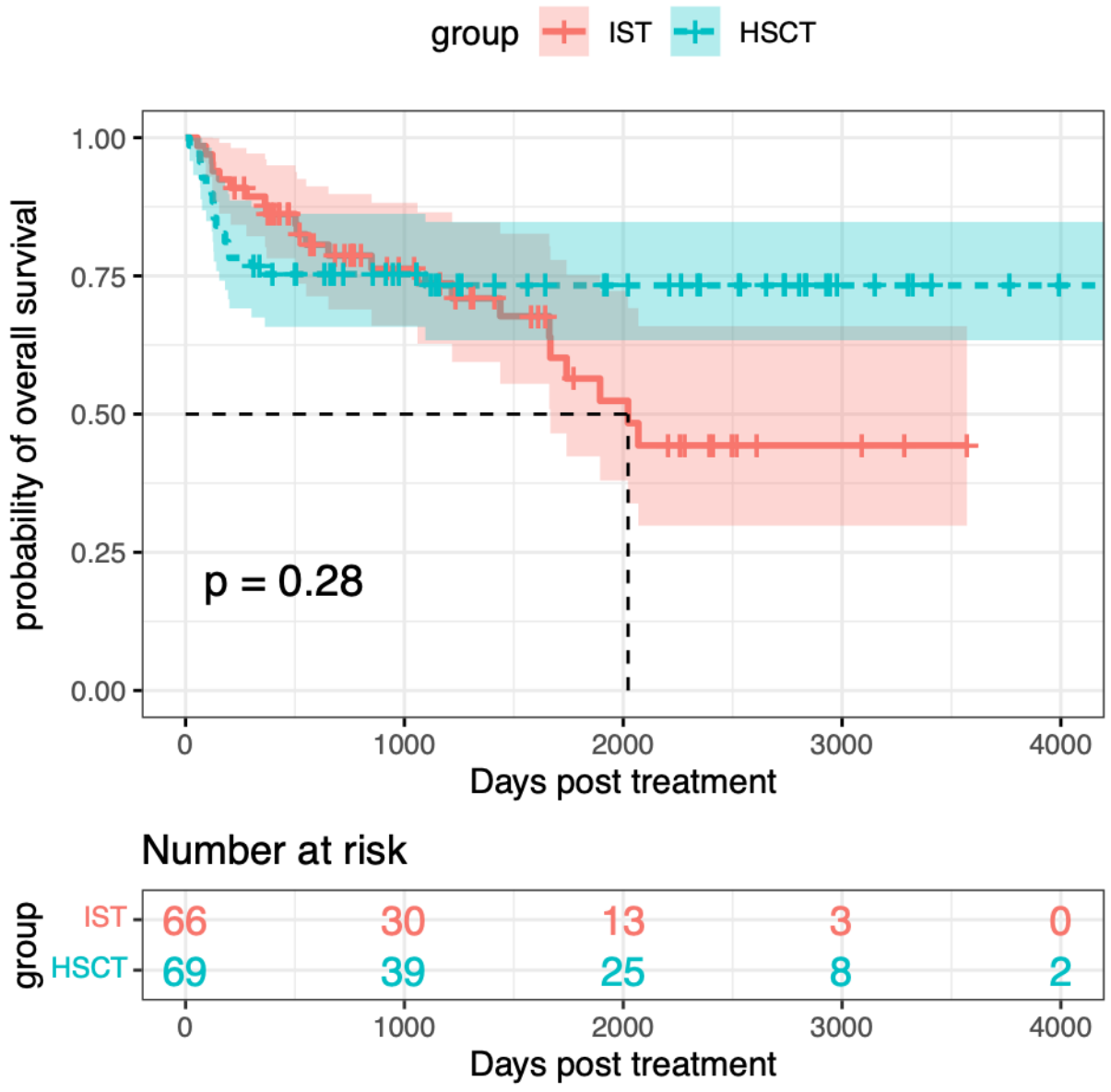

Supplementary Figure 1. Overall survival of IST vs. HID HSCT Cohort Before Propensity Score Matching (n=66 vs 69, P = 0.28)

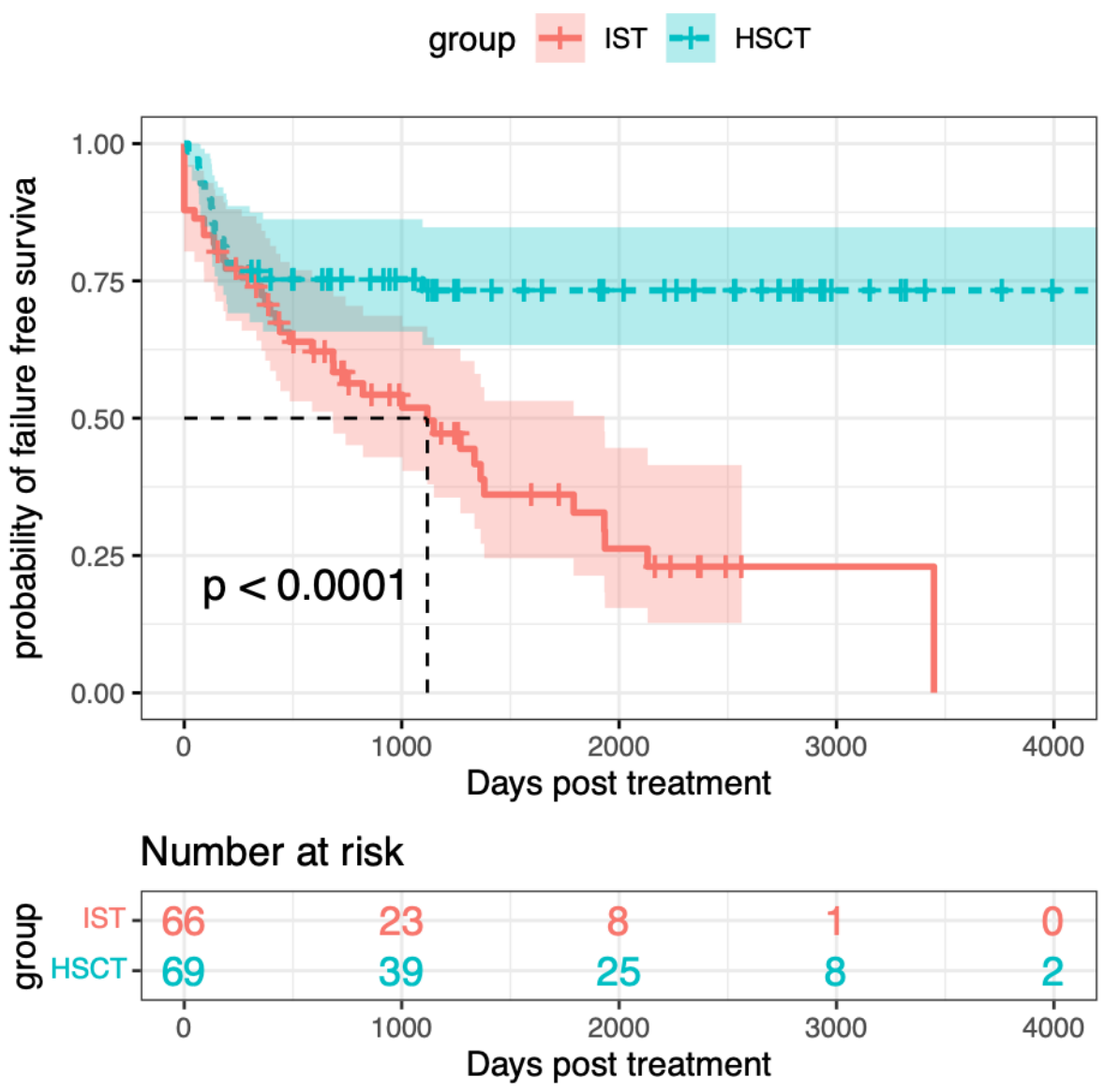

Supplementary Figure 2. Failure free survival of IST vs. HID HSCT Cohort Before Propensity Score Matching (n=66 vs 69, P <0.0001)
